# Serum Fetuin-A levels are increased and associated with insulin resistance in women with polycystic ovary syndrome

**DOI:** 10.1186/s12902-020-0538-1

**Published:** 2020-05-19

**Authors:** Sha Liu, Wenjing Hu, Yirui He, Ling Li, Hua Liu, Lin Gao, Gangyi Yang, Xin Liao

**Affiliations:** 1 Department of Endocrinology, the Affiliated Hospital, Zunyi Medical University, Zunyi, 563003 Guizhou China; 2 Chongqing Prevention and Treatment Hospital for Occupational Diseases, Chongqing, China; 3 Department of Endocrinology, the Second Affiliated Hospital, Chongqing Medical University, Chongqing, China; 4 Key Laboratory of Diagnostic Medicine (Ministry of Education) and Department of Clinical Biochemistry, College of Laboratory Medicine, Chongqing Medical University, Chongqing, 400016 China; 5 Department of Pediatrics, University of Mississippi Medical Center, 2500 North State Street, Jackson, MS USA

**Keywords:** Fetuin-a, Polycystic ovary syndrome, Insulin resistance

## Abstract

**Background:**

Insulin resistance (IR) is a common characteristic of women with polycystic ovary syndrome (PCOS). It has been reported that circulating Fetuin-A levels were associated with IR and type 2 diabetes mellitus (T2DM). However, previous reports were inconsistent.

**Methods:**

Two hundred seven subjects were screened for PCOS according to the diagnostic guideline of the Rotterdam consensus criterion. Serum Fetuin-A levels were measured using an ELISA kit. An independent *t-*test or Nonparametric test was used to detect differences between PCOS and control groups. Spearman’s correlation analysis was used to examine the association of the serum Fetuin-A with other parameters.

**Results:**

Our findings showed that circulating Fetuin-A concentration ranged from 196.6 to 418.2 μg/L for most women without PCOS (95%). Women with PCOS had higher circulating Fetuin-A levels than healthy women (437.9 ± 119.3 vs. 313.8 ± 60.5 μg/L; *p* <  0.01). Serum Fetuin-A was positively correlated with BMI, WHR, TG, TC, LDL-C, HOMA-IR, LH, T, and DHEA-S. Multivariate regression analysis showed that WHR, TG, HOMA-IR, and DHEA-S were independent predictors of the levels of circulating Fetuin-A. Binary logistic regression revealed that serum Fetuin-A was associated with the occurrence of PCOS. In addition, our ROC curve analysis found that the cutoff values for Fetuin-A to predict PCOS and IR were 366.3 and 412.6 μg/L.

**Conclusion:**

Blood Fetuin-A may be a useful biomarker for screening women for PCOS and IR.

## Background

Polycystic ovary syndrome (PCOS) is one of the most common endocrine, metabolic diseases in adolescent women. It has three main characteristics: oligo or amenorrhea (OA), hyperandrogenism (HA), and/or clinical manifestations of HA, polycystic ovary (PCO), and most cases are accompanied by obesity and other metabolic disorders. In addition to symptoms caused by hyperandrogenism and reproductive disorders, increasing evidence supports the central role of insulin resistance (IR) and compensatory hyperinsulinemia in the pathogenesis of the PCOS [1]. Furthermore, women with PCOS have an increased risk of developing other metabolic diseases, such as obesity, dyslipidemia, chronic inflammation, metabolic syndrome (MetS), type 2 diabetes mellitus (T2DM), atherosclerosis, and cardiovascular disease (CVD) [2, 3]. Overall, IR affects up to 70% of women with PCOS. Despite extensive research, the mechanisms underlying IR in PCOS patients are not entirely understood [4].

The pathogenesis of PCOS is complex, and its etiology remains unclear. From the definition of PCOS to its phenotype, heterogeneity is an inherent feature of PCOS, and its formation also has heterogeneity. In different phenotypes of PCOS, the relative contribution of excessive androgen and other factors, such as obesity and IR, to the development of PCOS has also been manifested in different ways [5]. Although in the past few decades, many studies have explored the mechanism of metabolic disorders and IR in PCOS patients, the current diagnostic criteria do not include indicators reflecting metabolic disorders and IR [6–8]. Therefore, it is essential to look for circulating biomarkers that reflect metabolic disorders and IR in PCOS patients.

Alpha-2-Heremans-Schmid glycoprotein (Fetuin-A) is a 64 kDa glycoprotein and previously considered to be a hepatokine [9]. However, recently, some studies have found that adipose tissue can also express and secrete Fetuin-A [10, 11]. Therefore, Fetuin-A is defined as both a hepatokine and an adipokine. Previous studies have shown that Fetuin-A is related to glucose and lipid metabolism and IR, including 1) inhibition of insulin action through inhibition of the auto-phosphorylation of insulin receptor tyrosine kinase and glucose transporter 4 (GLUT4); 2) combination with saturated fatty acids may cause Fetuin-A to stimulate chronic inflammation through the Toll-like receptor 4 (TLR4), leading to IR [12–14]; 3) the mRNA and protein of Fetuin-A are increased in ob/ob mice [14]. 4) impairing of adipocyte function leads to IR [15]; 5) increased Fetuin-A expression is associated with endoplasmic reticulum (ER) stress leading to the development of IR [16]. In human studies, it has been found that polymorphisms in Fetuin-A were related to T2DM [17] and circulating Fetuin-A levels are elevated or decreased, or unchanged in obese patients, T2DM, non- alcoholic fatty liver disease (NAFLD), MetS, PCOS and CVD, and are either associated with or not associated with impaired glucose tolerance and IR [18–24]. These inconsistent findings on the relationship between Fetuin-A and IR, as well as metabolic diseases, necessitate further investigation.

In this study, we selected newly diagnosed women with PCOS as the subjects of research and evaluated the relationship between circulating Fetuin-A and IR in vivo.

## Methods

### Study population

This study was performed from December 2018 to September 2019. One hundred and twenty-two women with PCOS (PCOS group, 19–37 years old) and eighty-five normal controls (N group, 19–32 years old) participated in the current study. The diagnosis of PCOS was based on the Rotterdam consensus criterion, which met two of the following three criteria [25]: 1) hyperandrogenism and/or clinical manifestations of hyperandrogenism; 2) oligo or amenorrhea; 3) ultrasound imaging of polycystic ovary. Other related diseases and disorders were excluded. All PCOS patients were newly diagnosed without lifestyle intervention or any medication. Eighty-five age-matched women were recruited in this study as normal controls. Control subjects had normal menstrual cycles (21–35 days), normal progesterone (P4) level in the luteal phase, and had no acne, hair loss, or hirsutism. Their ovarian morphology was normal by ultrasonography. Exclusion criteria include T2DM, thyroid disease, CVD, liver, and kidney dysfunction. The subjects were recruited from outpatient clinics, daily physical examinations, or advertisements in schools or communities. All subjects signed informed consent before participating in the study. The study was approved by the Human Research Ethics Committee of ZunYi Medical University and performed in accordance with the Helsinki Declaration.

### Anthropometric measurement

Body mass index (BMI) was calculated as weight divided by height squared. Waist circumference and hip circumference were measured by the same observer for calculation of waist-to-hip ratio (WHR). The formula of homeostasis model assessment of IR (HOMA-_IR_) was fasting insulin (FIns, lU/ml) × fasting blood glucose (FBG, mmol/L)/22.5 [26]. According to HOMA-_IR_, individuals were divided into IR (HOMA-_IR_ ≥ 3.8) and non-IR (HOMA-IR < 3.8) [27]. After 12–14 h of fasting, blood samples were obtained from all the subjects before breakfast and centrifuged at 4 °C. The serum was stored at − 80 °C for further analysis.

### Measurements of serum Fetuin-a, sex hormone, and biochemical parameters

Blood glucose, HbA1c, insulin levels, and blood fat, including triglyceride (TG), total cholesterol (TC), low-density lipoprotein cholesterol (LDL-C), and high-density lipoprotein cholesterol (HDL-C) were measured as in a previous population [28].The electrochemiluminescence immunoassay was performed for measuring serum follicle-stimulating hormone (FSH), luteinizing hormone (LH), and testosterone (T) by using COBASE immunoassay analyzers (Roche Diagnostics GmbH). An automated analyzer was used for measuring dehydroepiandrostenedione sulfate (DHEA-S) [29].

The serum concentration of Fetuin-A was determined by enzyme-linked immunosorbent assay (ELISA) according to the manufacturer’s instructions. Both intra- and inter-assay variations were 10 and 8%, respectively. The measuring range of this kit was 9.38–600 ng/ml. For the determination of sex hormones, venous blood samples in the healthy controls were collected from day 3 to 5 of the menstrual cycle (early follicular period), and in women with PCOS, blood samples were collected after spontaneous bleeding or amenorrhea for more than 3 months [30].

#### Statistical analysis

Statistical analyses were performed by using SPSS software version 19.0 (SPSS, Chicago, IL). Results were presented as mean ± SD or median (interquartile range) unless indicated otherwise. The variables of a non-normal distribution were transformed through logarithm before analysis. An independent sample *t-*test or Nonparametric test was performed for comparisons between two groups. One-way ANOVA with post hoc analysis was used to investigate differences in body composition and other indicators between PCOS women and healthy controls. Simple and multiple linear regression analysis was used to study the correlation between fasting Fetuin-A concentrations and other biomarkers. The association of Fetuin-A with PCOS was assessed by multivariate logistic regression analysis. The sensitivity and specificity of Fetuin-A for predicting PCOS were evaluated by a receiver operating characteristic (ROC) curve. Sample size was calculated using the following equations: N = [Zα/2 σ/εμ]2 (σ, standard; μ, mean; Zα/2 = 1.96, α = 0.05, ε = 10%) .*p* <  0.05 was considered significant.

## Results

### Serum Fetuin-a concentration in PCOS and healthy women

**Fig. 1:**
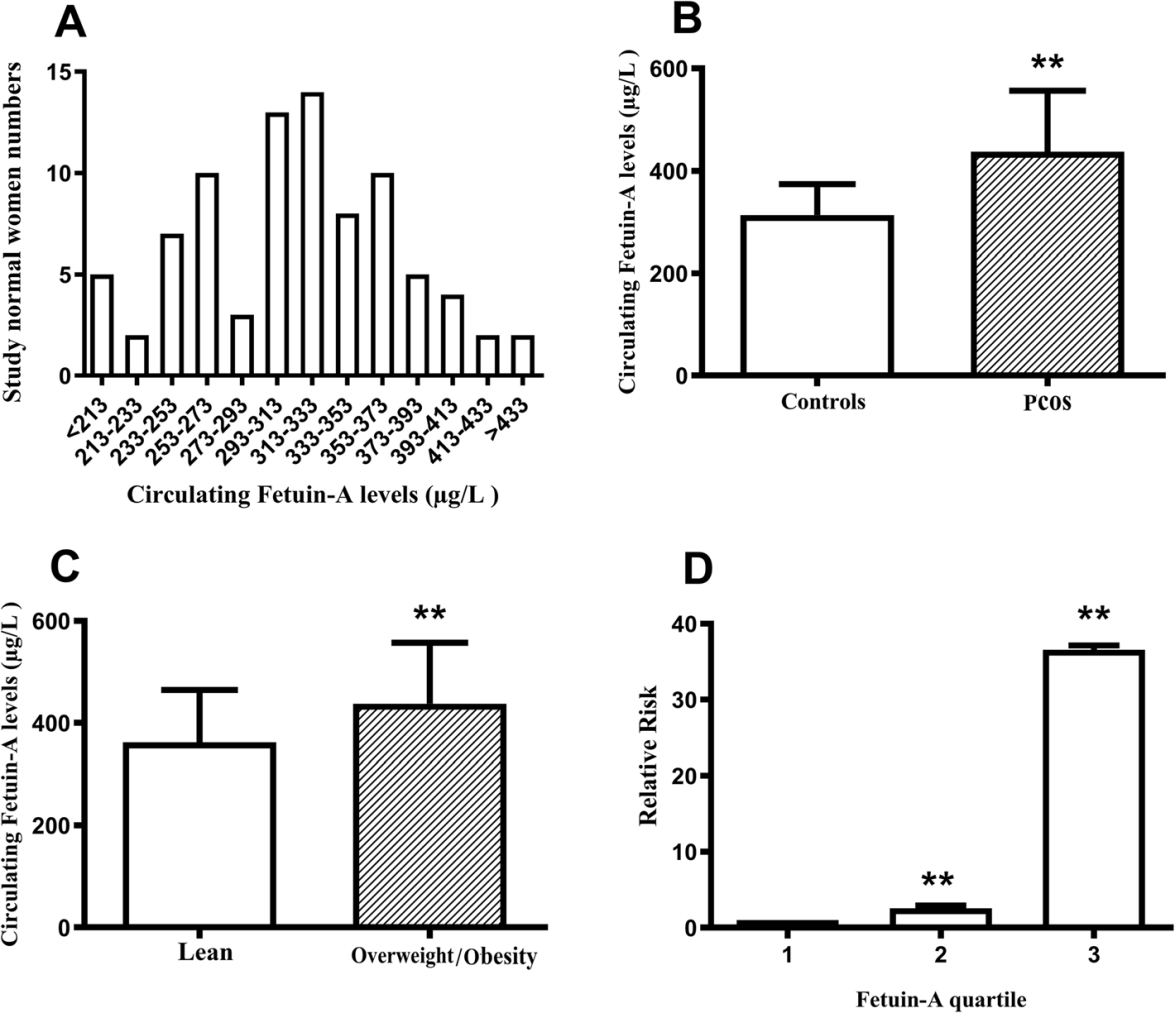
Serum Fetuin-A levels in the study population. **a** Distribution of circulating concentration of Fetuin-A in normal women. **b** Serum Fetuin-A levels in healthy and PCOS women. **c** Circulating Fetuin-A levels in lean and obese/overweight subjects. **d** The odds ratio of having MetS in different tertiles of circulating Fetuin-A. Data were expressed as means ± SD. *p < 0.05, ** *p* < 0.01 vs. Controls, lean or tertile 1

Table 1 summarized the demographic, anthropometric, and metabolic parameters and sex hormone levels of all women in the current study. The distribution of Fetuin-A concentrations in healthy women was shown in Fig. 1a. We found that circulating Fetuin-A concentration ranged from 196.6 to 418.2 μg/L for most healthy women (95%). PCOS patients had higher circulating Fetuin-A levels than healthy women (Fig. 1b, Table 1). PCOS patients were divided into obese/overweight (ob/ow) and lean groups by BMI < 25 kg/m^2^ or ≥ 25 kg/m^2^, respectively. In all study population, we found that the levels of Fetuin-A in obese/overweight group were significantly higher than those in the lean group (Fig. 1c). When healthy women were divided into overweight and lean groups, there was no significant difference in serum Fetuin-A (313.5 ± 60.6 vs. 317.4 ± 62.7 μg/L), suggesting that there was no significant relationship between serum Fetuin-A level and overweight in healthy women. Furthermore,

**Table 1:** Main clinical features and circulating Fetuin-A levels in PCOS and healthy women

Characteristics	Controls (*n* = 85)	PCOS (*n* = 122)	*p*-value
Age (yr)	26.0 ± 3.4	25. 3 ± 3.4	0.114
BMI (kg/m^2^)	21.6 ± 2.9	24.4 ± 4.4	< 0.001
WHR	0.81 ± 0.08	0.88 ± 0.10	< 0.001
SBP (mmHg)	113 ± 8	128 ± 8	< 0.001
DBP (mmHg)	75 ± 7	77 ± 7	0.045
TG (mmol/L)	0.92 (066–1.34)	1.37(1.01–2.48)	< 0.001
TC (mmol/L)	3.94 ± 0.76	4.64 ± 0.93	< 0.001
HDL-C (mmol/L)	1.23 ± 0.27	1.18 ± 0.33	0.278
LDL-C (mmol/L)	2.18 ± 0.58	2.89 ± 0.58	< 0.001
FBG (mmol/L)	4.77 ± 0.46	5.06 ± 0.91	< 0.01
FIns (mU/L)	7.40 (5.55–9.17)	18.00 (9.30–27.08)	< 0.001
HOMA-_IR_	15.0 (1.15–2.04)	3.85 (1.97–5.83)	< 0.001
FSH (IU/L)	5.92 ± 2.00	5.78 ± 1.36	0.574
LH (IU/L)	6.07 ± 5.14	8.39 ± 4.94	< 0.001
T (nmol/L)	1.01 ± 0.68	1.44 ± 0.65	< 0.001
DHEA-S (μmol/L)	4.50 ± 1.86	6.58 ± 2.93	< 0.001
Fetuin-A (μg/L)	313.8 ± 60.5	437.9 ± 119.3	< 0.001

Serum Fetuin-A was divided into three concentrations (quantile 1 < 322.6 μ g / L; quantile 2.

322.6–419.6 μg/L; quantile 3 > 419.6 μ g / L). The results showed that the incidence of PCOS was significantly higher in the quantile 2 and 3 than in the quantile 1 (95% CI 1.28–5.14 in quantile 2 and 95% CI 11.8–113.5 in quantile 3; Fig. 1d).

Values are given as mean ± SD or median (inter quartile range). BMI, Body mass index; WHR, Waist hip ratio; SBP, Systolic blood pressure; DBP, Diastolic blood pressure; FBG, fasting blood glucose; FIns, fasting plasma insulin; TG, Triglyceride; TC, Total cholesterol; HDL-C, High-density lipoprotein cholesterol; LDL-C, Low-density lipoprotein cholesterol; HOMA-IR, homeostasis model assessment of insulin resistance; FSH, follicle-stimulating hormone; LH, luteinizing hormone; T, testosterone; DHEA-S, dehydroepiandrosterone sulfate.

### Serum Fetuin-a level and its association with other parameters in the study population

Next, we investigated the relationship between the levels of circulating Fetuin-A and various other parameters. Serum Fetuin-A was positively correlated with BMI, WHR, TG, TC, LDL-C, HOMA-_IR_, LH, T, and DHEA-S (Table 2). Moreover, as previously reported, we also found a significant correlation between T and HOMA-_IR_ (*r* = 0.351, *p* <  0.01). We then performed a multiple stepwise regression to determine variables that had independent associations with serum Fetuin-A. The results showed that only WHR, TG, HOMA-_IR,_ and DHEA-S were independent predictors of the levels of circulating Fetuin-A (Table 2). The multiple regression equation was Y _lg10(Fetuin-A)_ = 2.11 + 0.012 X_HOMA-IR_ + 0.401X_WHR_ + 0.017X_TG_+ 0.009X _DHEA-S_ .

**Table 2:** Correlation analysis of variables associated with circulating Fetuin-A levels in the study populations

	Simple		Multiple	
Variable	*r*	*p*	*b*	*p*
Age (years)	−0.124	0.076	–	–
BMI (kg/m^2^)	0.294	< 0.001	–	–
SBP (mmHg)	0.076	0.279	–	–
DBP (mmHg)	0.039	0.579	–	–
WHR	0.351	< 0.001	0.401	< 0.001
TG (mmol/L)	0.293	< 0.001	0.017	< 0.05
TC (mmol/l)	0.184	< 0.01	–	–
HDL-C (mmol/L)	- 0.106	0.128	–	–
LDL-C (mmol/L)	0.339	< 0.001	–	–
HOMA-_IR_	0.511	< 0.001	0.012	< 0.001
FSH (IU/L)	- 0.079	0.259	–	–
LH (IU/L)	0.178	< 0.05	–	–
T (nmol/L)	0.320	< 0.001	–	–
DHEA-S (μmol/L)	0.330	< 0.001	0.009	< 0.01

Fetuin-A concentration was a non-normal distribution and transformed by logarithm before analysis. Additionally, logistic regression analysis revealed that Fetuin-A was significantly related to PCOS, even after controlling for anthropometric variables, blood lipid and so on (Table 3).

**Table 3:** Association of circulating Fetuin-A with PCOS in fully adjusted models

Model adjustments	PCOS		
	OR	95%CI	*P*
Age, SBP, DBP	1.015	1.010–1.020	0.000
Age, SBP, DBP, BMI, WHR	1.014	1.009–1.019	0.000
Age, SBP, DBP, BMI, WHR, FBG, FIns	1.011	1.005–1.017	0.000
Age, SBP, DBP, BMI, WHR, FPG, FIns, Lipid profile	1.010	1.004–1.017	0.002
Age, SBP, DBP, BMI, WHR, FPG, FIns, Lipid profile, Hormone parameters	1.008	1.001–1.016	0.036

*CI* confidence interval; *OR* odds ratio. Logistic regression was used to analyze the data of two groups

### ROC curve analysis

**Fig. 2:**
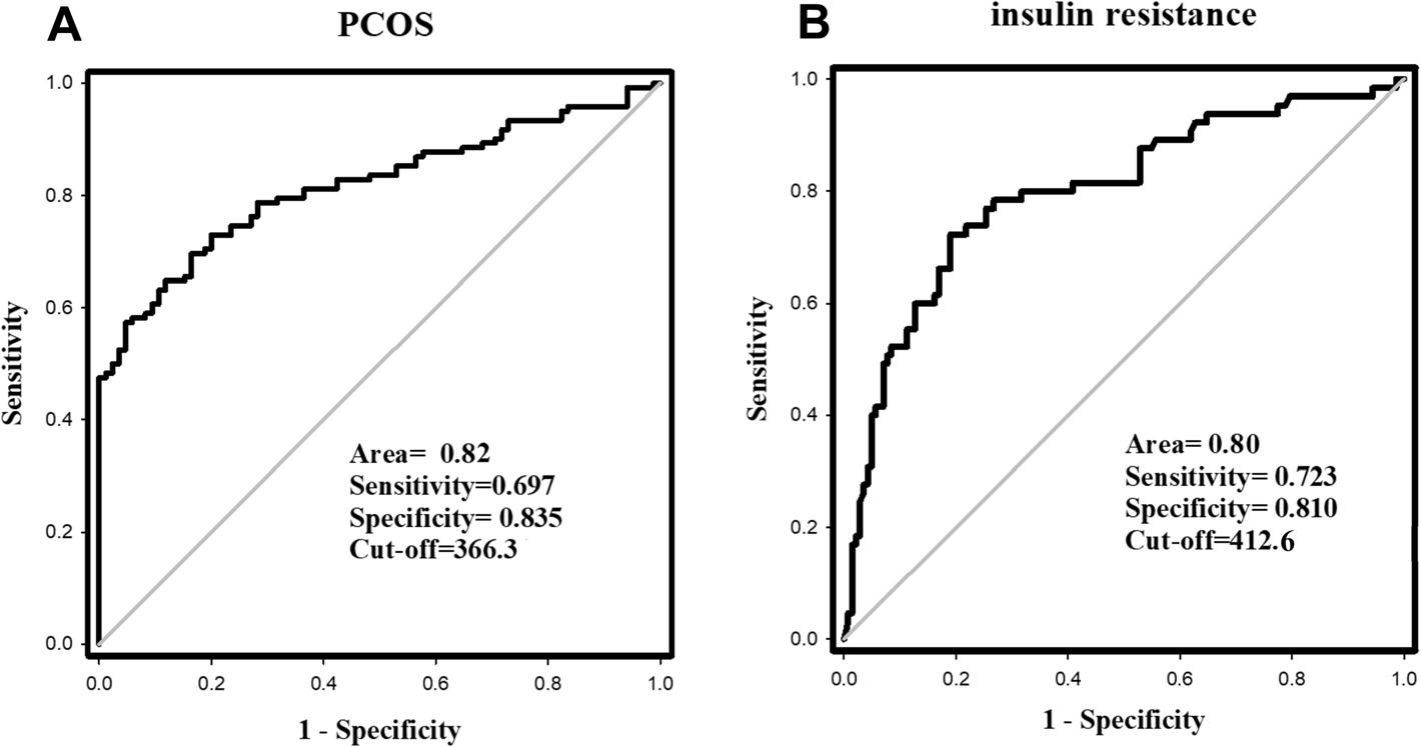
**a** ROC curve analysis of the prediction of PCOS. **b** ROC curve analysis of the prediction of insulin resistance

To explore the prediction of PCOS and IR by blood Fetuin-A, we performed a ROC curve analysis. The results showed that the area under the ROC curves for PCOS (AUC_PCOS_) was 0.82 with a specificity of 83.5%, and sensitivity of 69.7% (*p* < 0.01, Fig. 2a), and AUC_IR_ was 0.80 with a specificity of 81%, and a sensitivity of 72.3%. The best cut-off values for Fetuin-A to detect PCOS and IR were 366.3 μg/L, and 412. 6 μg/L, respectively.

## Discussion

In the past decade, several population-based studies reported the relationship between circulating Fetuin-A concentrations and PCOS. However, the conclusions of these studies were contradictory. In previous studies, circulating Fetuin-A levels were increased, decreased, or unchanged in PCOS patients compared with healthy women [22, 31–35]. In the current investigation, we found that circulating Fetuin-A concentrations were markedly elevated in women with PCOS compared with healthy women. Our results were consistent with those of Enli et al. [33–35] but contrary to those of Díaz et al. [22]. The cross-sectional nature of the study and population heterogeneity might be related to differences in outcomes. Furthermore, previous studies had small sample sizes, and some had no healthy controls or did not include obese patients with PCOS. In this study, we avoid these shortcomings. In addition, we employed newly diagnosed PCOS women to avoid the effects of medication, lifestyle interventions, and the duration of the disease that were associated with those patients who were under treatment. The reason for the rise in circulating Fetuin-A was unknown. We speculate that the metabolic disorders and hyperandrogenism caused by IR (hyperinsulinemia) may promote the synthesis and release of Fetuin-A in vivo. In addition, increased Fetuin-A might be derived, at least in part, by the status of low-grade inflammation, since inflammatory cytokines, such as CRP, were increased in women with PCOS.

Two previous studies found that Fetuin-A inhibited the insulin receptor tyrosine kinase and Toll-like receptor 4 in liver and muscle cells to suppress insulin signaling and stimulate inflammatory signaling pathways [13, 14]. However, population-based studies showed that there was no correlation between Fetuin-A and IR in diabetic patients, and there was no correlation between Fetuin-A and the risk of diabetes [36, 37]. In the current work, we found that serum Fetuin-A was positively correlated with BMI, WHR, TG, TC, LDL-C, HOMA-_IR_, LH, T, and DHEA-S, suggesting that Fetuin-A was associated with hyperinsulinemia and hyperandrogenism. These data supported the results of Pal et al. and Srivas et al. [13]. Because circulating Fetuin-A levels were associated with both T and IR, and hyperandrogenism and IR were essential characteristics of PCOS, it was of clinical significance to consider Fetuin-A a biomarker for PCOS.

Surprisingly, serum Fetuin-A concentration increased significantly in obese/overweight PCOS women but did not change in normal women with obese/overweight. This result was consistent with a previous study [38]. The reason for this was unknown. We speculate that the main factors affecting circulating Fetuin-A might be hyperinsulinemia and hyperandrogenemia, not adipose mass. In our study cohort, a small number of overweight women might affect the results. In addition, under hyperinsulinemia and hyperandrogenism, elevated circulating Fetuin-A raised the question of whether lowering Fetuin-A concentrations were the key to improving IR and hyperandrogenism. Secondly, because serum Fetuin-A levels were related to hyperandrogenemia, it was important to observe whether circulating Fetuin-A concentration would change with the menstrual cycle due to the change of hormone levels. To address these questions, further study is necessary.

We analyzed the ROC curve to explore the best cut-off point for predicting PCOS with circulating Fetuin-A. Our results showed that cyclic Fetuin-A was a good predictor for PCOS patients. Thus, we consider that the association of Fetuin-A with PCOS may be due to the high incidence of IR in PCOS population.

Our research also has some limitations. Firstly, the study population is young women, so our results may not apply to an elderly population. Secondly, our results are based on a single measurement of Fetuin-A. Without repeated measures at different time points, the introduction of random measurement errors in determining biochemical variables is possible. Finally, the nature of the cross-sectional study makes it impossible for our results to explain the causal relationship between increased Fetuin-A levels and the occurrence of IR and PCOS.

## Conclusion

In conclusion, our results suggested that serum Fetuin-A levels were increased in PCOS patients. Circulating Fetuin-A concentrations were associated with dyslipidemia, IR, and ovarian hyperandrogenism in women with PCOS.

## Data Availability

The datasets used and/or analyzed during the current study are available from the corresponding author on reasonable request.

## Abbreviations

PCOS: Polycystic ovary syndrome
IR: Insulin resistance
T2DM: Type 2 diabetes mellitus
BMI: Body mass index
MetS: Metabolic syndrom
WHR: Waist-to-hip ratio
TG: Triglyceride
TC: Total cholesterol
LDL-C: Low-density lipoprotein cholesterol
HDL-C: High-density lipoprotein cholesterol
HOMA-IR: Homeostasis model assessment of insulin resistance
FBG: Fasting blood glucose
LH: Luteinizing hormone
FSH: Follicle-stimulating hormone
T: Testosterone
DHEA-S: Dehydroepiandrostenedione sulfate
SBP: Systolic blood pressure
DBP: Diastolic blood pressure
FIns: Fasting plasma insulin
CI: Confidence interval
OR: Odds ratio
ROC: Receiver operating characteristic
